# Divergence of premating behaviors in the closely related species *Drosophila subquinaria* and *D. recens*

**DOI:** 10.1002/ece3.477

**Published:** 2013-01-09

**Authors:** Erin M Giglio, Kelly A Dyer

**Affiliations:** Department of Genetics, University of GeorgiaAthens, GA, 30602

**Keywords:** Mate choice, olfaction, premating isolation, reproductive isolation

## Abstract

Most animal species use distinctive courship patterns to choose among potential mates. Over time, the sensory signaling and preferences used during courtship can diverge among groups that are reproductively isolated. This divergence of signal traits and preferences is thought to be an important cause of behavioral isolation during the speciation process. Here, we examine the sensory modalities used in courtship by two closely related species, *Drosophila subquinaria* and *Drosophila recens,* which overlap in geographic range and are incompletely reproductively isolated. We use observational studies of courtship patterns and manipulation of male and female sensory modalities to determine the relative roles of visual, olfactory, gustatory, and auditory signals during conspecific mate choice. We find that sex-specific, species-specific, and population-specific cues are used during mate acquisition within populations of *D. subquinaria* and *D. recens*. We identify shifts in both male and female sensory modalities between species, and also between populations of *D. subquinaria*. Our results indicate that divergence in mating signals and preferences have occurred on a relatively short timescale within and between these species. Finally, we suggest that because olfactory cues are essential for *D. subquinaria* females to mate within species, they may also underlie variation in behavioral discrimination across populations and species.

## Introduction

Courtship rituals allow individuals to identify and choose among potential mates. Premating courtship signals usually involve stereotyped motions, chemical pheromones or excretions, visual displays, or a combination of these signals (Greenspan and Ferveur 2000). These signal traits and preferences can be targets of sexual selection within species to ensure the highest level of fitness possible for an individual's offspring (Andersson 1994). Courtship rituals can also vary between isolated groups, and thus play a role in species recognition to allow individuals to identify and avoid mating with individuals of other species (Groening and Hochkirch 2008). Because behavioral isolation is often the main component of reproductive isolation between recently diverged taxa, divergence of signal traits and preferences are thought to be an important cause of isolation during the speciation process (Coyne and Orr 2004).

The courtship behaviors of some *Drosophila* flies have been well characterized, particularly in the model species *D. melanogaster* (Spieth 1974; Greenspan and Ferveur 2000). *Drosophila* integrate several different sensory modalities during courtship (Krstic et al. 2009), which are involved in both species recognition and sexual selection. For example, these can include visual wing displays and auditory wing songs (Blyth et al. 2008), visual information (Spieth 1974), gustatory and tactile signals that are sensed through tapping and licking (Everaerts et al. 2010), and chemical pheromones, which are sensed through olfactory pathways (Amrein 2004). The relative importance of particular cues may differ between the sexes (Ferveur 2010). Across species, the relative importance of each cue can differ, such that a particular cue may be essential to the courtship of one species while being entirely absent from the courtship behavior of another (Markow and O'Grady 2005).

Here, we examine the sensory modalities used during successful courtship and mate acquisition in two closely related *Drosophila* species that are incompletely reproductively isolated. *D. subquinaria* and *D. recens* are in the quinaria group of the subgenus Drosophila, and are common in the boreal forests of western and eastern North America, respectively. Their ranges overlap in central Canada for a region of about 1200 km (Jaenike et al. 2006); based on their biogeographic distributions, *D. subquinaria* and *D. recens* were probably isolated at the last glacier maximum and came back into sympatry during the last 20,000 years. These species are morphologically indistinguishable except for the male genitalia, and have no sex-specific pigmentation patterns (Wheeler 1960). Where they co-occur, they can be found at the same mushrooms, which serve as a mating substrate as well as a food source for both larvae and adults.

Behavioral studies show that *D. recens* females will occasionally mate with *D. subquinaria*, with no difference in female discrimination between populations that are sympatric and allopatric with *D. subquinaria* (Jaenike et al. 2006). In contrast, *D. subquinaria* females show a pattern of reproductive character displacement: females from populations sympatric with *D. recens* almost never mate with *D. recens* males, whereas *D. subquinaria* females from outside this region of overlap mate with *D. recens* males at a moderate rate. Furthermore, there is also behavioral isolation within *D. subquinaria*, as females from populations sympatric with *D. recens* also discriminate against conspecific *D. subquinaria* males from allopatric populations, with no known post-zygotic isolation (Jaenike et al. 2006). A potential selective force for these patterns of behavioral discrimination is a *Wolbachia* infection in *D. recens*: the offspring of *D. subquinaria* females and *D. recens* males die during embryogenesis, whereas in the reciprocal cross, the offspring survive and the F1 daughters are fertile (Shoemaker et al. 1999). Hybrid males are always sterile (Shoemaker et al. 1999).

In this study, we investigate the importance of different sensory modalities during conspecific mate selection in *D. subquinaria* and *D. recens*. We also ask whether the courtship pattern and sensory modalities are the same in sexually isolated populations of *D. subquinaria*. We use observational methods to characterize successful courtship patterns, and manipulative methods to remove various sensory modalities in males and/or females to determine the importance of each modality on mating success with conspecifics. We find differences in sensory modalities between *D. subquinaria* and *D. recens*, as well as between populations of *D. subquinaria*, which are consistent with strong divergent selection on premating behavior on sympatric *D. subquinaria*. Our results suggest that changes in courtship and sensory modalities used in courtship can occur on a very rapid evolutionary timescale.

## Materials and Methods

### Fly stocks and maintenance

We used *D. recens* collected in 2009 in Peru, New York; this population is allopatric to *D. subquinaria*, and is representative of other sympatric and allopatric populations of *D. recens* (K. A. Dyer, unpubl. data). There is no sexual isolation and very little genetic differentiation among populations of *D. recens* (Jaenike et al. 2006; K. A. Dyer, unpubl. data). We used flies from two populations of *D. subquinaria* in this study, including a representative allopatric and sympatric population with respect to the geographic range overlap with *D. recens*. Allopatric *D. subquinaria* were collected near Deary, Idaho, in 2009. This population was selected because there is substantial gene flow with the sympatric region and the flies show an ‘allopatric’ phenotype: females discriminate against *D. recens* at a much lower level than sympatric *D. subquinaria*, and they also do not discriminate against conspecific males from any population (E. R. Bewick & K. A. Dyer, unpubl. data). Sympatric *D. subquinaria* were collected near Edmonton, Alberta, in 2010. At the time of collection, the proportion of *D. subquinaria* and *D. recens* in this population was 8% and 92%, respectively, and *D. subquinaria* females from this population discriminate against *D. recens* as well as allopatric *D. subquinaria* (Bewick & K. A. Dyer, unpubl. data). Each population stock consisted of between five and ten isofemale lines that were allowed to interbreed for three generations before being used in experiments. All stocks were maintained in uncrowded conditions on Instant *Drosophila* food (Carolina Biological, Burlington, NC) supplemented with commercial mushroom (*Agaricus bisporus*). Cultures were kept at 20°C on a 14:10 light cycle and with 60% relative humidity. Light carbon dioxide anesthesia was used to collect virgins, which were stored at a density of 10–15 flies per vial.

### Courtship observations

During morning hours, one male and one female 7-day-old virgin from the same population were aspirated into a 2-cm diameter observation chamber containing a blended mushroom-agar media covered with a moistened piece of filter paper. Using a HD video camera (Canon Vixia HFG10; Canon USA, Lake Success, NY), we recorded the flies' behavior for 20 min or until copulation commenced. We did not record behaviors that occurred during the copulation, nor did we record the copulation duration. Later, the video was observed and any activity following the male orienting on the female was recorded, as was the overall courtship time. We scored the duration of orienting, tapping, and licking, and the frequency of circling, wing vibrations, and wing extensions. We note that males can display multiple behaviors simultaneously, all of which were scored. The data are based on 25 successfully copulating pairs from each population. Data were analyzed using Kruskal-Wallis (K-W) nonparametric tests and post-hoc comparisons of the means using the nonparametric Steel-Dwass (S-D) method. All statistical analyses were completed using JMP version 9.0.2 (SAS Institute, Cary, NC).

### Manipulative sensory analyses

Between 2 and 5 days after eclosion, virgin flies were placed under light carbon dioxide anesthesia and randomly assigned to either a control (unmanipulated) or experimental (manipulated) group. Experimental manipulations included blinding or removing the aristae, antennae, or wings. To obscure vision, we dabbed metallic paint from a paint marker (Elmer's Painters brand) over the flies' eyes. To render flies deaf, we removed the aristae, which is the feathery structure attached to each antenna, as close to the base as possible with microscissors. Sound waves vibrate the arista, and then a mechanical receptor in the antenna converts the vibrations into auditory perception (Göpfert and Robert 2002). To remove the flies' ability to smell, we removed the antennae between the second and third segments with surgical forceps. The third segment of the antenna is a primary location where receptors used for smell (olfaction) reside (Carlson 1996; Hallem et al. 2006; Vosshall and Stocker 2007). Note that removing the antenna also removes the arista, which is attached to the third segment of the antenna. Finally, we removed the wings at the base with a surgical scalpel. Wings can be used in multiple sensory modalities; for example, different species use them to create auditory songs, to flash visual cues, and to fan pheromones (Markow and O'Grady 2005; Gleason et al. 2012). Thus, the wing removal is not simply the ablation of a single sensory modality. In our study, we have not explicitly tested whether each sensory ablation actually removed the targeted modality from these species; instead, we assume that the underlying physiology and neurobiology of sense perception is similar across *Drosophila* species and that functional studies in *D. melanogaster* are applicable to other species.

Control flies were handled in the same ways and at the same times as the experimental flies, but were not surgically manipulated. All flies were placed in fresh food vials to recover. Seven to 11 days after emergence, including at least 5 days after manipulation, a single virgin male and female from the same species and population were placed together by aspiration into a fresh food vial. Four types of crosses were simultaneously set up for each manipulation: control male x experimental female, experimental male × control female, experimental male × experimental female, control male × control female. These pairs were left together for either 3 or 24 h, whereupon the male was removed by aspiration and discarded. Vials where either fly died during the mating trial were discarded. Two weeks later, each vial was checked for the presence of offspring, thereby indicating whether mating occurred. In this way, we assayed 30 pairs of flies for each cross type and modality manipulation for each population. Because the various manipulations were not all completed at the same time, data were analyzed separately for each manipulation and species/population. We used logistic regression by general linearized model (GLM with binomial error distribution and logit link) to assess the effect of each treatment (male type, female type, interaction) on mating rate. We found no test with a significant interaction term (all *P* > 0.1); thus, we repeated the analyses removing the interaction effect, and we report these models in the results.

## Results

### Behavioral observations

**Figure 1:**
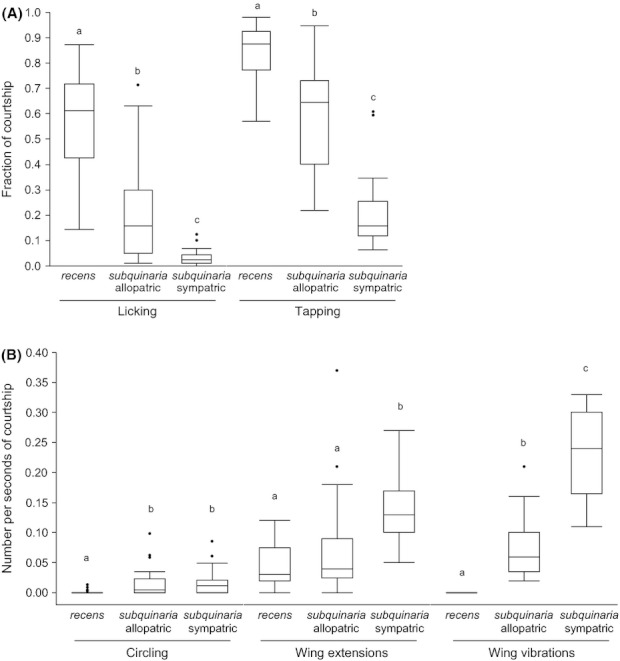
Box plot of the incidence of each specific behavior displayed during courtship, with results shown for each population. Part A) shows the proportion of courtship time males spent licking and tapping the female, and part B) shows the frequency of wing movements and circling behaviors per second of courtship. Post-hoc tests were completed separately for each behavior, as described in the text, and significant groupings are indicated with letters.

We will first describe the general courtship pattern for each species and population we studied; these are also summarized in Fig. 1, and we have included example videos as supplemental material. Then, we will compare quantitatively the incidence of specific courtship behaviors across species and populations. The typical *D*. *recens* courtship pattern begins with the male orienting to the female. He then taps her rapidly on the abdomen with his foremost pair of feet. Often at this point, the female moves away, to which the male responds by chasing and tapping her. Either way, the male quickly advances to simultaneously licking the female's genitals and tapping her abdomen near the genitalia with both of his first set of legs. This tapping is very fast and often repeated; if the male is close enough to the female to be tapping her during courtship, he constantly does so. Some males (16.0%) used a circling behavior around the female during this period of tapping. This was always followed by more tapping and licking, after which the male quickly extended one wing outward horizontally repeatedly at intervals of about 1 sec between extensions. On average, a courtship contained a median of seven wing extensions; usually only the same wing is extended, although either wing can be used. We never observed any wing vibrations by *D. recens* males. Eventually, the female spreads her wings and copulation occurs. On average, flies that copulated took 489 ± 68 [mean ± SE] sec to achieve copulation, of which 315 ± 53 sec were spent with the male oriented on the female. Overall, 25 of 43 (58%) pairs copulated within the 20-min observation window.

In the allopatric *D. subquinaria*, courtship begins with male orientation on the female. This is followed by the male tapping, and if the female moves away, by chasing. After the initial tapping, the male begins wing motions. In these males, wing vibration is more common than wing extensions (median of five wing extensions and seven wing vibrations per courtship), and all males displayed both of these behaviors. Most males (60%) then proceed to circling, which is often followed by wing motion. If the male can reach the female, he will then extend his proboscis and lick the female's genitals. Wing vibration is particularly common during genital licking. After licking, the male attempts to copulate; if the female is receptive, she spreads her wings and the male mounts and copulation begins. On average, flies that copulated took 449 ± 50 sec to achieve copulation, of which 185 ± 36 sec were spent with the male oriented on the female. Overall, 25 of 43 (58%) pairs copulated within the 20-min observation window.

Courtship of the sympatric *D. subquinaria* was largely similar to allopatric *D.* *subquinaria*. Males begin by orienting on the female, followed by tapping and chasing and then wing motions. As with allopatric *D.* *subquinaria*, wing motions are comprised of a mixture of wing vibrations and wing extensions, with a median of 15 wing extensions and 29 vibrations per courtship. Again as in allopatric *D. subquinaria*, circling then occurs in most (60%) males, followed by additional wing movement. Genital licking usually follows, and is accompanied by wing motion, and then females signal receptivity by spreading wings, and males respond by mounting and copulating. On average, pairs that mated took 430 ± 63 sec to achieve copulation, of which 240 ± 54 sec were spent with the male oriented on the female. Overall, 25 of 48 (52%) pairs copulated within the 20-min observation window.

Considering only flies that copulated during the observation period, the total amount of time it took pairs to copulate from when they were placed in the vial did not vary significantly across populations/species (K-W χ^2^ = 0.7, d.f. = 2, *P* = 0.7). The time to copulation from when the male oriented on the female also did not vary significantly among populations/species (K-W χ^2^ = 4.9, d.f. = 2, *P* = 0.09). During courtship (defined as any time in which the male is oriented on the female), we observed many differences in the amount of various behaviors displayed, both between species and between populations of *D.* *subquinaria*. For example, the proportion of time spent in tapping and licking during courtship varies significantly among the three populations (Fig. 1A; tapping: K-W χ^2^ = 55, d.f. = 2, *P* < 0.0001; licking: K-W χ^2^ = 51, d.f. = 2, *P* < 0.0001). The means of each pair of populations are also significantly different from each other for each trait, with *D. recens* exhibiting the most and sympatric *D. subquinaria* the least (S-D tests all *P* < 0.0001). For a male, during tapping and licking, the male receives gustatory signals from the female; for a female, these male behaviors are tactile signals from the male. Differences among populations and species suggest that gustatory signals to the male be more important during courtship of *D. recens* and allopatric *D.* *subquinaria*. Consistent with this, tactile signals may be important to *D. recens* and allopatric *D. subquinaria* females and/or perhaps inhibitory to female sympatric *D. subquinaria*.

Wing motions also differed between species and populations. *D. recens* only engaged in wing extensions, never wing vibrations (Fig. 1B). In contrast, every *D. subquinaria* male from both allopatric and sympatric populations engaged in both wing extensions and wing vibrations. On average, allopatric *D. subquinaria* males displayed about as many wing extensions per second of courtship as *D. recens*, and sympatric *D. subquinaria* displayed about three times as many of this behavior per second of courtship as either of the other two populations (Fig. 1B; K-W χ^2^ = 29, d.f. = 2, *P* < 0.0001; S-D tests: *subq* sym vs. *recens Z* = 5.1, *P* < 0.0001; *subq* sym vs. *subq* allo *Z* = 3.9, *P* = 0.0002; *subq* allo vs. *recens Z* = 1.0, *P* = 0.55). There were also significant differences in the number of wing vibrations per second of courtship displayed by males from the three populations, with sympatric *D. subquinaria* displaying the most vibrations and *D. recens* the least (Figure 1B; K-W χ^2^ = 66, d.f. = 2, *P* < 0.0001; S-D tests: all *P* < 0.0006). Results were consistent when the total number of behaviors per courtship rather than unit of courting time was considered (results not shown).

Finally, we observe the male circling behavior in both species, although circling appears to be more frequent in *D. subquinaria* (Figure 1B). Allopatric and sympatric *D. subquinaria* displayed about the same number of circles per second of courtship, whereas *D. recens* displayed significantly fewer (Figure 1B; K-W χ^2^ = 15, d.f. = 2, *P* = 0.0006; S-D tests: *subq* sym vs. *recens Z* = −3.6, *P* = 0.001; *subq* allo vs. *recens Z* = 3.6, *P* = 0.002; *subq* sym vs. *subq* allo *Z* = 0.32, *P* = 0.9). The circling behavior was often in conjunction with wing movements, and thus may be a visual cue or a mechanism to emphasize other signals.

### Manipulative sensory analyses

**Figure 2:**
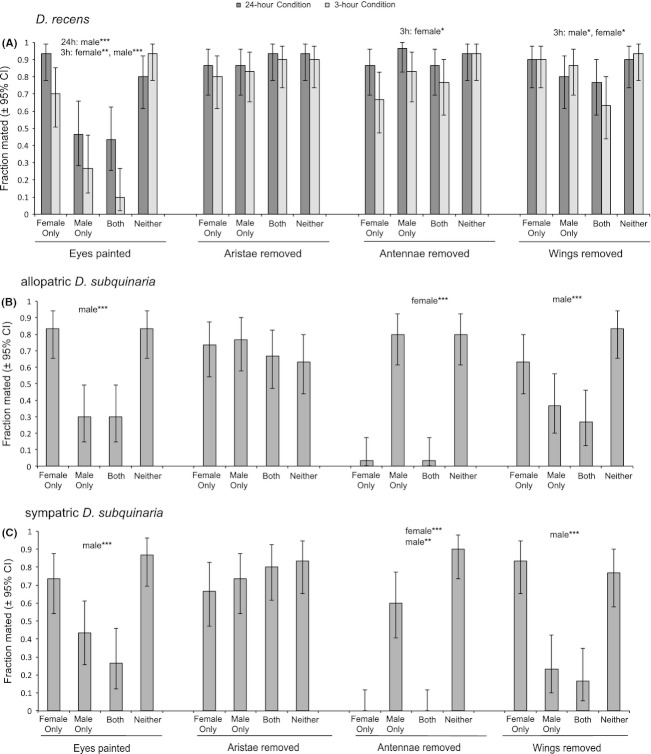
Mating success rates under different sensory modality manipulations. Shown are the results for A) *Drosophila recens*, B) allopatric *Drosophila subquinaria*, and C) sympatric *D. subquinaria*. “Female only” indicates only the female in that cross was altered, “Male only” indicates only the male was altered, etc. *N* = 30 crosses for each manipulation, and error bars indicate 95% confidence intervals, calculated with a binomial distribution. For *D. recens*, the dark and light gray bars show the results for 24-h and 3-h mating assays, respectively. For *D. subquinaria*, all mating assays lasted for 24 h. When the male or female effect is significant for a manipulation, significance values are indicated by the asterisks, with *P* < 0.05, *P* < 0.01, and *P* < 0.001 indicated by *, **, and ***, respectively.

The results of the sensory manipulations are summarized in Figure 2 and in Table S1. When *D. recens* males and females were kept together for 24 h, we observed no decrease in mating for any manipulation except when the male was blind (male effect: χ^2^ = 24, d.f. = 1; *P* < 0.0001). We repeated the entire experiment and shortened the time the male and female had access to each other to 3 h. In this shorter window, both male and female vision were very important for mating success (male effect: χ^2^ = 56, d.f. = 1; *P* < 0.0001; female effect: χ^2^ = 8.4, d.f. = 1; *P* = 0.0038). Removal of the female's antennae resulted in a ∼30% decrease in mating (female effect: χ^2^ = 5.3, d.f. = 1; *P* = 0.021), and because removing of the female's aristae had no significant effect (female effect: χ^2^ = 0.1, d.f. = 1; *P* = 0.8), this reduction in mating is likely driven by lack of olfactory cues to the female. The presence of male wings was important for mating, and there was also a moderately significant effect on mating rate when females did not have wings (male effect: χ^2^ = 6.4, d.f. = 1; *P* = 0.011; female effect: χ^2^ = 4.1, d.f. = 1; *P* = 0.042). This may indicate that both sexes use wing-originating cues from the other sex. Thus, from this experiment, we can infer that in this population of *D. recens,* visual cues are important for males, and visual and olfactory cues are important for females to mate.

For the allopatric *D. subquinaria,* significant decreases in mating frequency were observed in the 24-h condition for several sensory modality losses (Fig. 2b; Table S1). Again, male vision was important to mating, as male blindness decreased mating rate about 65% relative to the control (male effect: χ^2^ = 37, d.f. = 1; *P* < 0.0001), and for this species, this effect was not seen for female blindness (female effect: χ^2^ = 0, d.f. = 1; *P* = 1.0). Removal of the male antennae, and thus both olfactory and auditory cues, did not affect mating (male effect: χ^2^ = 0, d.f. = 1; *P* = 1.0). However, removal of the female antennae decreased mating rate by 95% (female effect: χ^2^ = 85, d.f. = 1; *P* < 0.0001), such that when females were without antennae, the pairs almost never mated. Because removing the aristae of either sex had no effect for mating success (male effect: χ^2^ = 0.2, d.f. = 1; *P* = 0.7; female effect: χ^2^ = 0, d.f. = 1; *P* = 1.0), this suggests that removing the female's antennae eliminates essential olfactory cues that are critical for females to mate. Male winglessness also significantly affected mating success (male effect: χ^2^ = 22, d.f. = 1; *P* < 0.0001), which is likely due to the females refusing males that do not produce a signal produced by the wings. We completed a small 3-h manipulation focusing only on winglessness for allopatric *D. subquinaria*, and the effects were identical to the 24-h manipulation: male winglessness was found to decrease mating success by 60%, but female winglessness had no effect (male effect: χ^2^ = 20, d.f. = 1; *P* < 0.0001; female effect: χ^2^ = 2.1, d.f. = 1; *P* = 0.14). In summary, for these allopatric *D. subquinaria*, visual cues are important for males, but wing-originating and especially olfactory cues are critical for females to mate.

The sensory modalities used by the sympatric *D.* *subquinaria* were similar to their allopatric counterparts in most, but not all, respects (Fig. 2c; Table S1). As for the allopatric *D. subquinaria*, loss of male vision reduced male mating by 50% (male effect: χ^2^ = 27, d.f. = 1; *P* < 0.0001), with a small effect on mating when females were blind (female effect: χ^2^ = 3.5, d.f. = 1; *P* = 0.06). Strikingly, the loss of female antennae reduced mating to zero (female effect: χ^2^ = 96, d.f. = 1; *P* < 0.0001); of 60 pairs where the female's antennae were removed, not a single pair mated in the 24-h time frame. Because female aristae were again unimportant for mating (female effect: χ^2^ = 0.4, d.f. = 1; *P* = 0.5), this suggests that chemosensory cues are critical for females to mate. Interestingly, when the male sympatric *D. subquinaria* antennae were removed, there was also a reduction in mating – about 35% compared with the control (male effect: χ^2^ = 7.6, d.f. = 1; *P* < 0.006). Because the removal of the male aristae had no effect on mating success (male effect: χ^2^ = 0.04, d.f. = 1; *P* = 0.8), this suggests that male *D. subquinaria* from sympatric populations may use olfactory cues in courtship. This pattern is in contrast to male allopatric *D. subquinaria* and *D. recens*, which show no effect on mating of removing their antennae or aristae (all *P* = 1.0). When males were without wings, mating was reduced by 60% (male effect: χ^2^ = 46, d.f. = 1; *P* < 0.0001); again, this may be because the signals the wings produce were absent from courtship, which caused the male to not be accepted by the female. In summary, our manipulative study suggests that for these sympatric *D. subquinaria*, visual and olfactory cues are important for males to mate, and wing-originating and olfactory cues are critical for females to mate.

## Discussion

The typical *Drosophila* courtship is a complex series of behaviors, and signals can include auditory, visual, gustatory, olfactory, and tactile cues (reviewed in Greenspan and Ferveur 2000). In this study, we used both observational and manipulative experiments to determine which sensory modalities are involved in courtship and mate acquisition within *D. recens* and *D.* *subquinaria*. Observational studies are useful to determine the specific behaviors that the male and female display. Sensory manipulation experiments that ablate sensory modalities can provide insight into the signaling modes used by each sex as well as species and population differences (e.g., Gleason et al. 2012). In addition, ablation experiments can indicate whether mate acceptance depends on a compound signal that uses more than one type of sensory modality. Our goal in this study is to ask whether the same sensory modalities are important for mating within populations of *D. subquinaria* and *D. recens*.

From our manipulative study, we found that vision was important for males of both *D. subquinaria* and *D. recens*. This is a common result across *Drosophila* (Markow and O'Grady 2005), and it is thought to be the case that eyesight is necessary for proper orientation of the male to the female and for the male to be able to follow the female (Spieth 1974). Usually, the importance of vision is assayed by placing flies in the dark; however, this does not allow one to determine whether the male, female, or both sexes use visual cues as mating signals. By sex-specific ablation of vision, we were also able to show that vision is not only important for males but is also important for *D. recens* females to mate, although it does not appear to be essential as this effect is overcome when flies are allowed a longer period to mate (Fig. 2).

Both our observational and manipulative studies indicate that chemosensory signals are very important in these species. *D. recens* and allopatric *D. subquinaria* males spend most of their courtship tapping and licking the females (Fig. 1A). Because both the *Drosophila* proboscis and the forelegs have gustatory receptors (Amrein and Thorne 2005), and because the removal of the male antennae did not decrease male mating frequency in either of these types of males (Fig. 2), this suggests that the bulk of chemosensory information for these males may be conveyed through contact gustatory signaling rather than through olfaction. In other species, gustatory signals have been shown to be important for male mating behavior (Watanabe et al. 2011). These behaviors may also provide important tactile cues to the females, and we cannot disentangle the importance of these behaviors for male versus female mating based only on observational studies. In contrast to male allopatric *D. subquinaria* and *D. recens*, we find that the male sympatric *D. subquinaria* appear to place more emphasis on olfactory than gustatory signals. Not only did sympatric *D. subquinaria* males without antennae mate less frequently than control males with antennae (Fig. 2), but in our observational studies, these males also displayed fewer gustatory behaviors than the other populations (Fig. 1). From these data, it appears that a shift in sensory modality from gustatory to olfactory signals has occurred in male sympatric *D.* *subquinaria*. We speculate that selection may have acted on these sympatric males to increase discrimination for species recognition, perhaps through olfactory pathways. The caveat of this is that we cannot test whether these males are actually receiving fewer gustatory signals, only that they display the behavior less. It is also possible that a shift in the preference of sympatric females has occurred, such that these tactile cues have become inhibitory when they exceed a certain threshold. Further study with additional populations is necessary to determine the geographic extent of this difference in male behavior, in particular whether it correlates with the zone of geographic overlap between species. It will also be important to determine whether these males court females from other populations with less vigor than their own females.

Chemosensory cues are also very important for females of both of the species we studied. For female *D. recens*, there is a significant effect of removing the female's antennae, but the mating rate only drops to about 65% in 3 h, with no significant effect over a longer 24-h period (Fig. 2; Table S1). In contrast, female *D. subquinaria* are critically dependent on olfactory cues, regardless of population and even after a 24-h mating period. Olfactory signals have been shown to be very important for mating in many other species of *Drosophila* (Ferveur 2005) and for insects more generally (Howard and Blomquist 2005). In particular, in *Drosophila,* pheromones are involved in both species and mate recognition, and have been shown to evolve rapidly and be targets of selection (Chenoweth and Blows 2005; Rundle et al. 2005; Grillet et al. 2006; Van Homrigh et al. 2007; Higgie and Blows 2008). Given the critical importance of these signals to conspecific mating, olfactory cues are obvious candidates to investigate as potential signals in species and/or population-level discrimination in *D.* *subquinaria*.

Finally, both our observational and manipulative experiments suggest the role of the male's wings during courtship differs between species. *D. recens* males display only wing extensions, while *D. subquinaria* males display both wing extensions and vibrations during courtship, and there is a significant effect of ablating the male's wings in both species (Table S1, Fig. 2). However, for both species, removing the female's aristae to render her deaf does not decrease the mating rate. Male wing motions are known to be important in *Drosophila* courtship signaling, and the specific sensory signaling involved in wing motion can differ across species (Lasbleiz et al. 2006). The presence of wing vibrations may create a song in *D. subquinaria*, and in other species of *Drosophila* male song can vary within populations and differ between closely related vspecies, including within the quinaria group of *Drosophila* (Ewing and Miyan 1986; Hoikkala et al. 1994; Ritchie et al. 1994; Neems et al. 1997; Colegrave et al. 2000; Routtu et al. 2007; Turner and Miller 2012). However, given the lack of an effect of ablating the aristae, it may be more likely that in these species, the wings are involved in creating non-auditory signals to the female. In many species, the male's wings create visual signals (Yeh et al. 2006), and in some species, the male's wings fan pheromones to the female (Gleason et al. 2012). Non-auditory signals would be consistent with the findings discussed above that vision is important for female mating in *D. recens*, and that olfaction is important for females of both species, especially *D. subquinaria*. For example, if there has been an increase in reliance on olfaction in *D. subquinaria* females, and if males of this species are using their wings to fan pheromones, the wing requirement would be consistent with this change. Further investigation is necessary to disentangle the role of the wings during courtship, and the presence of a song must be assessed, especially in *D. subquinaria*.

Summing across our results, it is clear that sex-specific, species-specific, and population-specific cues are used during mate acquisition within populations of *D. recens* and *D. subquinaria*. In addition, it appears that mate acceptance is not dependent entirely on one sensory modality, but that multi-modal signals are often at play. For example, in male *D. recens* and allopatric *D. subquinaria*, both visual and chemosensory modalities appear to be important, although further experiments are needed to test the combined effect of these modalities. Likewise, in female *D. recens*, both visual and olfactory cues are necessary for mating. The exception to this may be within female *D. subquinaria*, where only olfactory signals are essential for mating. In other *Drosophila* species, often two or more sensory modalities must be ablated together in order to completely reduce mating success, indicating that multiple cues are necessary for successful mating (e.g., Gailey et al. 1986). It is much rarer to find a single sensory modality that, upon ablation, completely abolishes mating success (but see Gleason et al. 2012). We note that because we find differences among populations and species in the consequences of sensory ablation on mating, this suggests that it is not just the injury itself that leads to our results, but that our data uncover real biological patterns.

The sensory signaling systems that are important for mate choice within species are also likely to be involved in mate discrimination between populations and/or species (Andersson 1994; Price 1998; Panhuis et al. 2001; Svensson and Gosden 2007). Knowing which behavioral sensory modalities are used within populations will help to build a comprehensive understanding of how selection causes them to diverge and thus contribute to the speciation process. Using both observation and manipulative studies, we find broad-scale divergence in courtship pattern and sensory modalities between the closely related species *D. subquinaria* and *D. recens*. We also find significant differences between two populations of *D. subquinaria*, which is particularly interesting because these populations experience gene flow and are geographically not very distant from each other. Further work is necessary to determine the extent to which courtship behaviors vary throughout the range of *D. subquinaria*, but the differences found here are consistent with the observed pattern of reproductive character displacement in female discrimination (Jaenike et al. 2006). Analyzing whether the signal varies in a pattern similar to the discrimination behavior displayed by the females will be a key to determining which signals are involved in reinforced mate discrimination between species and among populations. In other words, one would expect character displacement in the male signal in a pattern concordant with that of the female preferences. On the basis of our results, here we suggest that olfactory signals, for example, cuticular hydrocarbons, are the obvious first place to look, but we also suggest that the role of the wings needs to be investigated further.

## Supporting Information

**Table S1.** Mating frequency and results of a logistic regression for each manipulation.

**Video S1.** Example video recording of Allopatric *D. recens* courtship.

**Video S2.** Example video recording of Allopatric *D. subquinaria* courtship.

**Video S3.** Example video recording Sympatric *D. subquinaria* courtship.

## References

[b1] Amrein H (2004). Pheromone perception and behaviour in *Drosphila*. Curr. Opin. Neurobiol.

[b2] Amrein H, Thorne N (2005). Gustatory perception and behavior in *Drosophila melanogaster*. Curr. Biol.

[b3] Andersson M (1994). Sexual Selection.

[b4] Blyth JE, Lachaise D, Ritchie MG (2008). Divergence in multiple courtship song traits between *Drosophila santomea* and *D. yakuba*. Ethology.

[b5] Carlson JR (1996). Olfaction in Drosophila: from odor to behavior. Trends Genet.

[b6] Chenoweth SF, Blows MW (2005). Contrasting mutual sexual selection on homologous signal traits in *Drosophila serrata*. Am. Nat.

[b7] Colegrave N, Hollocher H, Hinton K, Ritchie MG (2000). The courtship song of African *Drosophila melanogaster*. J. Evol. Biol.

[b8] Coyne JA, Orr HA (2004). Speciation.

[b9] Everaerts C, Lacaille F, Ferveur JF (2010). Is mate choice in *Drosophila* males guided by olfactory or gustatory pheromones?. Anim. Behav.

[b10] Ewing A, Miyan JA (1986). Sexual selection, sexual isolation and the evolution of song in the *Drosophila repleta* group of species. Anim. Behav.

[b11] Ferveur JF (2005). Cuticular hydrocarbons: their evolution and roles in *Drosophila* pheromonal communication. Behav. Genet.

[b12] Ferveur JF (2010). *Drosophila* female courtship and mating behaviors: sensory signals, genes, neural structures and evolution. Curr. Opin. Neurobiol.

[b13] Gailey DA, Lacaillade RC, Hall JC (1986). Chemosensory elements of courtship in normal and mutant, olfaction-deficient *Drosophila melanogaster*. Behav. Genet.

[b14] Gleason JM, Pierce AA, Vezeau AL, Goodman SF (2012). Different sensory modalities are required for successful courtship in two species of the *Drosophila willistoni* group. Anim. Behav.

[b15] Göpfert MC, Robert D (2002). The mechanical basis of *Drosophila* audition. J. Exp. Biol.

[b16] Greenspan RJ, Ferveur JF (2000). Courtship in *Drosophila*. Annu. Rev. Genet.

[b17] Grillet M, Dartevelle L, Ferveur JF (2006). A *Drosophila* male pheromone affects female sexual receptivity. Proc. R. Soc. B.-Biol. Sci.

[b18] Groening J, Hochkirch A (2008). Reproductive interference between animal species. Q. Rev. Biol.

[b19] Hallem EA, Dahanukar A, Carlson JR (2006). Insect odor and taste receptors. Annu. Rev. Entomol.

[b20] Higgie M, Blows MW (2008). The evolution of reproductive character displacement conflicts with how sexual selection operates within a species. Evolution.

[b21] Hoikkala A, Kaneshiro KY, Hoy RR (1994). Courtship songs of the picture-winged *Drosophila planitibia* subgroup species. Anim. Behav.

[b22] Howard RW, Blomquist GJ (2005). Ecological, behavioral, and biochemical aspects of insect hydrocarbons. Annu. Rev. Entomol.

[b23] Jaenike J, Dyer KA, Cornish C, Minhas MS (2006). Asymmetrical reinforcement and *Wolbachia* infection in *Drosophila*. PLoS Biol.

[b24] Krstic D, Boll W, Noll M (2009). Sensory integration regulating male courtship behavior in *Drosophila*. PLoS ONE.

[b25] Lasbleiz C, Ferveur JF, Everaerts C (2006). Courtship behaviour of *Drosophila melanogaster* revisited. Anim. Behav.

[b26] Markow TA, O'Grady PM (2005). Evolutionary genetics of reproductive behavior in *Drosophila*: connecting the dots. Annu. Rev. Genet.

[b27] Neems RA, Dooher K, Butlin RK, Shorrocks B (1997). Differences in male courtship song among species of the *quinaria* group of *Drosophila*. J. Insect Behav.

[b28] Panhuis TM, Butlin R, Zuk M, Tregenza T (2001). Sexual selection and speciation. Trends in Ecol. and Evol.

[b29] Price T (1998). Sexual selection and natural selection in bird speciation. Phil. Trans. R. Soc. of Lond. Ser. B Biol. Sci.

[b30] Ritchie MG, Yate VH, Kyriacou CP (1994). Genetic variability of the interpulse interval of courtship song among some European populations of *Drosophila melanogaster*. Heredity.

[b31] Routtu J, Mazzi D, Mirol K, van der Linde P, Butlin R, Hoikkala A (2007). The extent of variation in male song, wing and genital characters among allopatric *Drosophila montana* populations. J. Evol. Biol.

[b32] Rundle HD, Chenoweth SF, Doughty P, Blows MW (2005). Divergent selection and the evolution of signal traits and mating preferences. PLoS Biol.

[b33] Shoemaker DD, Katju V, Jaenike J (1999). Wolbachia and the evolution of reproductive isolation between *Drosophila recens* and *Drosophila subquinaria*. Evolution.

[b34] Spieth HT (1974). Courtship behavior in *Drosophila*. Annu. Rev. Entomol.

[b35] Svensson EI, Gosden TP (2007). Contemporary evolution of secondary sexual traits in the wild. Funct. Ecol.

[b36] Turner TL, Miller PM (2012). Investigating natural variation in drosophila courtship song by the evolve and resequence approach. Genetics.

[b37] Van Homrigh A, Higgie M, McGuigan K, Blows MW (2007). The depletion of genetic variance by sexual selection. Curr. Biol.

[b38] Vosshall LB, Stocker RE (2007). Molecular architecture of smell and taste in Drosophila. Ann. Rev. Neurosci.

[b39] Watanabe K, Toba G, Koganezawa M, Yamamoto D (2011). Gr39a, a highly diversified gustatory receptor in *Drosophila*, has a role in sexual behavior. Behav. Genet.

[b40] Wheeler MR (1960). New species of the quinaria group of Drosophila (Diptera, Drosophilidae). Southwest. Nat.

[b41] Yeh SD, Liou SR, True JR (2006). Genetics of divergence in male wing pigmentation and courtship behavior between *Drosophila elegans* and *D. gunungcola*. Heredity.

