# Supplementary material for: Divergence of premating behaviors in the closely related species Drosophila subquinaria and D. recens
Source: Ecol Evol. 2013 Jan 9;3(2):365–74. doi: 10.1002/ece3.477 (PMC3586646; doi:10.1002/ece3.477)
Supplement: Supplementary file 1 [file ece30003-0365-SD1.pdf]

Supplemental Table 1. Mating frequency and results of a logistic regression for each manipulation.

Population	Time	Manipulation	Sex Manipulated	Total Pairs	Total Mated	Model/Effect	Likelihood ratio χ^2	d.f.	*P*-value
Allopatric recens	24 hrs	Eyes	Female Only	30	28	Whole model	24.82	2	**< 0.0001**
Allopatric recens	24 hrs	Eyes	Male Only	30	14	Female	0.41	1	0.520
Allopatric recens	24 hrs	Eyes	Both	30	13	Male	24.49	1	**< 0.0001**
Allopatric recens	24 hrs	Eyes	Neither	30	24				
Allopatric recens	24 hrs	Aristae	Female Only	30	26	Whole model	0	2	1
Allopatric recens	24 hrs	Aristae	Male Only	30	26	Female	0	1	1
Allopatric recens	24 hrs	Aristae	Both	30	28	Male	0	1	1
Allopatric recens	24 hrs	Aristae	Neither	30	28				
Allopatric recens	24 hrs	Antennae	Female Only	30	26	Whole model	3.47	2	0.176
Allopatric recens	24 hrs	Antennae	Male Only	30	29	Female	3.47	1	0.062
Allopatric recens	24 hrs	Antennae	Both	30	26	Male	0	1	1.00
Allopatric recens	24 hrs	Antennae	Neither	30	28				
Allopatric recens	24 hrs	Wings	Female Only	30	27	Whole model	3.19	2	0.203
Allopatric recens	24 hrs	Wings	Male Only	30	24	Female	0.064	1	0.800
Allopatric recens	24 hrs	Wings	Both	30	23	Male	3.128	1	0.077
Allopatric recens	24 hrs	Wings	Neither	30	27				
Allopatric recens	3 hrs	Eyes	Female Only	30	21	Whole model	60.406	2	**< 0.0001**
Allopatric recens	3 hrs	Eyes	Male Only	30	8	Female	8.389	1	**0.0038**
Allopatric recens	3 hrs	Eyes	Both	30	3	Male	55.574	1	**< 0.0001**
Allopatric recens	3 hrs	Eyes	Neither	30	28				
Allopatric recens	3 hrs	Aristae	Female Only	30	24	Whole model	0.137	2	0.934
Allopatric recens	3 hrs	Aristae	Male Only	30	25	Female	0.069	1	0.793
Allopatric recens	3 hrs	Aristae	Both	30	27	Male	0.069	1	0.793
Allopatric recens	3 hrs	Aristae	Neither	30	27				
Allopatric recens	3 hrs	Antennae	Female Only	30	20	Whole model	5.340	2	0.069
Allopatric recens	3 hrs	Antennae	Male Only	30	25	Female	5.340	1	**0.021**
Allopatric recens	3 hrs	Antennae	Both	30	23	Male	0	1	1.000
Allopatric recens	3 hrs	Antennae	Neither	30	28				

Allopatric recens	3 hrs	Wings	Female Only	30	27	Whole model	10.374	2	**0.006**
Allopatric recens	3 hrs	Wings	Male Only	30	26	Female	4.140	1	**0.042**
Allopatric recens	3 hrs	Wings	Both	30	19	Male	6.442	1	**0.011**
Allopatric recens	3 hrs	Wings	Neither	30	28				
Allopatric subquinaria	24 hrs	Eyes	Female Only	30	25	Whole model	36.845	2	< **0.0001**
Allopatric subquinaria	24 hrs	Eyes	Male Only	30	9	Female	0	1	1.000
Allopatric subquinaria	24 hrs	Eyes	Both	30	9	Male	36.845	1	< **0.0001**
Allopatric subquinaria	24 hrs	Eyes	Neither	30	25				
Allopatric subquinaria	24 hrs	Aristae	Female Only	30	22	Whole model	0.159	2	0.924
Allopatric subquinaria	24 hrs	Aristae	Male Only	30	23	Female	0	1	1.000
Allopatric subquinaria	24 hrs	Aristae	Both	30	20	Male	0.159	1	0.690
Allopatric subquinaria	24 hrs	Aristae	Neither	30	19				
Allopatric subquinaria	24 hrs	Antennae	Female Only	30	1	Whole model	85.421	2	< **0.0001**
Allopatric subquinaria	24 hrs	Antennae	Male Only	30	24	Female	85.421	1	< **0.0001**
Allopatric subquinaria	24 hrs	Antennae	Both	30	1	Male	0	1	1.000
Allopatric subquinaria	24 hrs	Antennae	Neither	30	24				
Allopatric subquinaria	24 hrs	Wings	Female Only	30	19	Whole model	24.851	2	< **0.0001**
Allopatric subquinaria	24 hrs	Wings	Male Only	30	11	Female	3.305	1	.069
Allopatric subquinaria	24 hrs	Wings	Both	30	8	Male	22.134	1	< **0.0001**
Allopatric subquinaria	24 hrs	Wings	Neither	30	25				
Allopatric subquinaria	3 hrs	Wings	Female Only	30	15	Whole model	22.696	2	< **0.0001**
Allopatric subquinaria	3 hrs	Wings	Male Only	30	7	Female	2.183	1	0.140
Allopatric subquinaria	3 hrs	Wings	Both	30	2	Male	20.837	1	< **0.0001**
Allopatric subquinaria	3 hrs	Wings	Neither	30	17				
Sympatric subquinaria	24 hrs	Eyes	Female Only	30	22	Whole model	29.423	2	< **0.0001**
Sympatric subquinaria	24 hrs	Eyes	Male Only	30	13	Female	3.519	1	0.061
Sympatric subquinaria	24 hrs	Eyes	Both	30	8	Male	26.649	1	< **0.0001**
Sympatric subquinaria	24 hrs	Eyes	Neither	30	26				
Sympatric subquinaria	24 hrs	Aristae	Female Only	30	20	Whole model	0.456	2	0.796
Sympatric subquinaria	24 hrs	Aristae	Male Only	30	22	Female	0.410	1	0.522
Sympatric subquinaria	24 hrs	Aristae	Both	30	24	Male	0.046	1	0.831
Sympatric subquinaria	24 hrs	Aristae	Neither	30	25				

Sympatric subquinaria	24 hrs	Antennae	Female Only	30	0	Whole model	98.890	2	**< 0.0001**
Sympatric subquinaria	24 hrs	Antennae	Male Only	30	18	Female	95.995	1	**< 0.0001**
Sympatric subquinaria	24 hrs	Antennae	Both	30	0	Male	7.595	1	**0.0059**
Sympatric subquinaria	24 hrs	Antennae	Neither	30	27				
Sympatric subquinaria	24 hrs	Wings	Female Only	30	25	Whole model	46.259	2	**< 0.0001**
Sympatric subquinaria	24 hrs	Wings	Male Only	30	7	Female	0	1	1.000
Sympatric subquinaria	24 hrs	Wings	Both	30	5	Male	46.259	1	**< 0.0001**
Sympatric subquinaria	24 hrs	Wings	Neither	30	23				
